# Compromised Refugia: Latitudinal Gradients Mimic Depth and Constrain Subtropical Reef Habitats in the Nilotic Basin

**DOI:** 10.1002/ece3.74367

**Published:** 2026-10-11

**Authors:** Anat Tsemel, Hagai Nativ, Stephane Martinez, Shai Einbinder, Ole Johannes Ringnander Sørensen, Yair Suari, Ido Izhaki, Dan Tchernov

**Affiliations:** ^1^ Kadas Nexus, Leon H. Charney School of Marine Sciences University of Haifa Haifa Israel; ^2^ Faculty of Marine Sciences Ruppin Academic Center Michmoret Israel; ^3^ Department of Evolutionary and Environmental Biology University of Haifa Haifa Israel

## Abstract

Upper‐mesophotic rocky reefs (25–30 m depth) have been proposed as climate refugia in the ultra‐oligotrophic Eastern Mediterranean for fish communities in warming shallow waters. A pronounced environmental gradient runs from north to south along the southeastern Levant, driven by sediment and nutrient discharge downstream of the receding Nile Delta. We hypothesized that higher surface productivity near the Nile Delta would support an algae‐dominated epibenthic food web and richer fish assemblages at the southern sites. We surveyed epibenthic cover, habitat structure, and fish assemblages at five sites spanning ~160 km of coastline within a shared 25–30 m depth band between 2017 and 2022. Contrary to this hypothesis, our findings reveal a distinct coastal ecocline, a benthic shift from algae‐ to bryozoan‐dominated epibenthos that closely mirrored the depth‐related shift observed at the northern sites (between 10 m and 45 m). This benthic shift was coupled with reduced abundance of herbivorous and micro‐invertivorous fish and with significantly lower fish species richness and diversity at the southern sites, despite their higher structural complexity. Redundancy analysis showed that depth, latitude, visibility, and season together explained 55.5% of the variance in epibenthic community composition, while an analogous spatial model explained 20% of the variance in fish abundance. This benthic shift, associated with gradients in depth, latitude, sedimentation, and light attenuation, appears to be a major architect of fish community structure in this region, rather than structural complexity. This implies that the southern Levant may act not as a corridor but as a restrictive filter, trapping species between warming surface waters and a deep‐sea‐like habitat that lacks the conditions required to support higher trophic levels; the capacity of upper‐mesophotic reefs to serve as thermal refugia is not uniform across space.

## Graphical abstract

Deep reefs (25–30 m) were expected to be climate havens for fish fleeing warming shallow waters near Egypt's Nile Delta—but the opposite was observed. As you head south, the reef habitat shifts from primary producers (algae) to filter‐feeding bryozoans, and fish abundance and diversity drop sharply. Turns out these “refuges” aren't one‐size‐fits‐all: some may actually trap fish in a habitat too poor to support them.

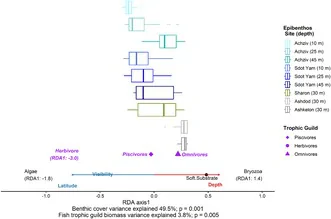

## 1 Introduction

The diversity of species and functions is deemed crucial for sustaining ecosystem resilience (Balvanera et al. 2006; Cardinale et al. 2006) particularly during periods of change (Hong et al. 2022). In the Eastern Mediterranean, an ultra‐oligotrophic “marine desert” (Azov 1991; Ben Ezra et al. 2021) undergoing unprecedented warming (Ozer et al. 2022), maintaining this biodiversity is a critical challenge. As shallow waters become increasingly hostile, upper‐mesophotic habitats are gaining recognition for their potential to serve as climate refugia (Tsemel et al. 2026). These deeper rocky reef zones host abundant, fishery‐targeted species and support greater fish biodiversity (Consoli et al. 2016; Pichot et al. 2025; Tsemel et al. 2026). However, understanding the environmental variables that shape these protective rocky reef ecosystems requires examining not only depth but also horizontal spatial gradients.

In coastal areas, abiotic factors such as nutrient availability, turbidity, reduced light levels, and sedimentation strongly restrict and shape marine communities (Maughan 2001; Irving and Connell 2002; Magris and Ban 2019). These light‐limiting processes are increasingly recognized as a global phenomenon: recent satellite‐based analyses indicate that the depth of the ocean's photic zone has contracted by more than 10% across over 9% of the global ocean since 2003, with darkening in coastal regions linked to increased sediment, nutrient, and organic‐matter loading (Davies and Smyth 2025).

Along the Levantine coast, a major horizontal gradient is driven by the receding Nile Delta. Longshore currents transport organic compounds and sediments eastward and northward (Abd‐El Monsef et al. 2015), increasing regional productivity but also altering the physical environment. Satellite imagery and seawater samples show higher Chlorophyll‐a concentration in the shallow coastal regions south of Tel Aviv compared to the northern coastline (Groom et al. 2005; D'Ortenzio and Ribera d'Alcalà 2009; Kress et al. 2019), and higher nutrient concentrations (Kress et al. 2019). Concurrently, there is a stark gradient in grain size; for example, silt content at a depth of 30 m is 43% at the southern Ashkelon site but drops to between 4.9% and 14% further north at Sdot Yam (Almogi‐Labin et al. 2012).

These distinct alterations in environmental conditions downstream of the Nile Delta, in an ultra‐oligotrophic system, probably dictate the structure of the rocky reef epibenthos (Brind'Amour et al. 2016), which forms the base of the food web. Changes at the benthic level inevitably cascade upward, shaping the composition and function of the fish community (Kennish 2001; Parsons et al. 2016). While some fish assemblages show increased abundance when exposed to nutrient enrichment (Grigg 1994; Guidetti et al. 2003; Azzurro et al. 2010), high turbidity can severely decrease both fish abundance and species richness (Russo 1989). Despite sediment disturbance being recognized as a major global threat to marine biodiversity across taxa and habitats (Magris and Ban 2019), the Eastern Mediterranean is essentially absent from this global evidence base, leaving the sediment sensitivity of this ultra‐oligotrophic system unresolved.

What are the processes that shape fish communities along the naturally occurring north‐to‐south gradient in the Eastern Mediterranean? We characterized the main abiotic parameters (e.g., habitat structure, light, and chlorophyll) and the resulting epibenthic cover (algae, invertebrate major groups, bare rock, and sediment) that shape fish communities in the Eastern Mediterranean.

Because increased productivity often supports richer ecosystems, we hypothesized that sites closer to the productive Nile Delta would be characterized by an epibenthic algae‐dominated food‐web base. We expected this base to support higher fish abundance and diversity than deeper or more distant sites, thereby enhancing localized ecosystem stability and resilience.

## 2 Materials and Methods

### 2.1 Study Sites and Experimental Design

Underwater rocky reefs and aeoline‐formed sandstone (Kurkar) occur along the Israeli Mediterranean shelf to depths exceeding 100 m (Neev and Ben‐Avraham 1977). Along the coastline, rocky reef habitats are available at depths of 25–30 m. However, shallower (10 m) and deeper (45 m) habitats are available only in the northern region.

To address latitudinal processes, we surveyed five sites distributed along approximately 160 km of coastline: Achziv, Sdot Yam, Sharon, Ashdod, and Ashkelon, focusing on the shared 25–30 m depth band (Figure 1). To assess depth‐related variability, shallower (10 m) and deeper (45 m) habitats were integrated into the experimental design at the northern Achziv and Sdot Yam sites, where they are widely available.

**FIGURE 1:**
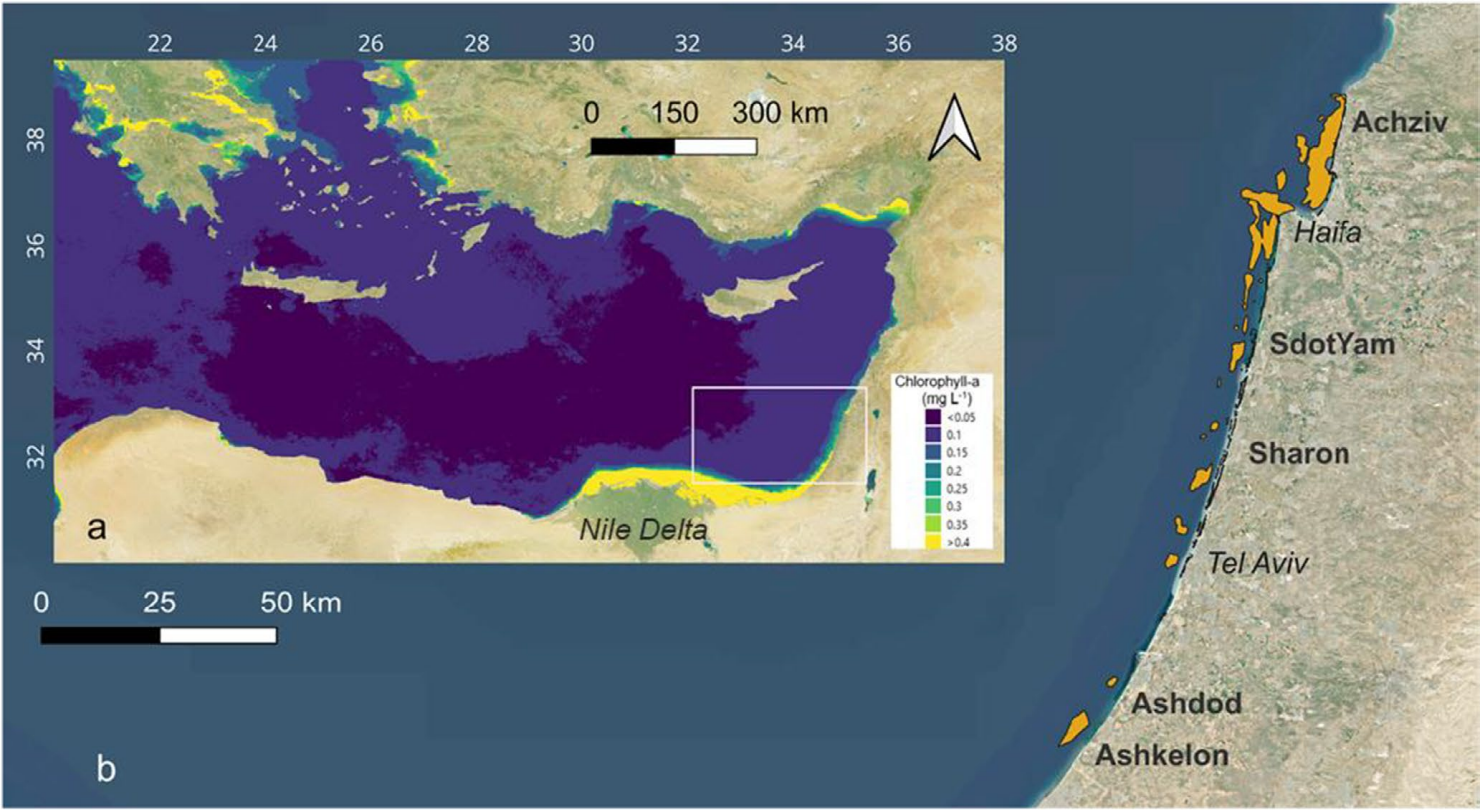
Map of the Levant Basin of the Mediterranean (a) and the south‐eastern Mediterranean coast (b; white inset box in a) with the 10 to 50 m depth shallow rocky reefs in yellow. The five survey sites are bold‐labeled. Remote‐sensing Chlorophyll‐a concentration from 10‐12‐2025 (Volpe et al. 2019) is projected onto (a).

Achziv, the northernmost site, is located on a submerged limestone ridge within a managed marine protected area (MPA). The remaining sites are unprotected and are atop Kurkar ridges, surrounded by soft sediments.

The 25 m and 45 m depth zones at Sdot Yam represent upper and lower mesophotic habitats, respectively (Tsemel et al. 2026). In this system, latitude acts as a reliable proxy for distance from the Nile Delta, dictating a long‐shelf environmental gradient documented by both remote sensing (Figure 1; Groom et al. 2005; D'Ortenzio and Ribera d'Alcalà 2009) and in situ observations (Almogi‐Labin et al. 2012; Kress et al. 2019).

### 2.2 Field Sampling and Environmental Measurements

The surveys took place between 2017 and 2022, targeting each site twice per year during periods when wave height is typically < 80 cm: spring (April—May) and fall (October–November). We censused four fixed, 25 m‐long belt transects per depth at identical locations across all sampling events; the 377 transects (Table A1) were conducted using Megalodon closed‐circuit rebreathers (CCR), which are safer and prevent scuba‐bubble‐induced behavioral biases.

Fish surveys were limited to conditions in which horizontal visibility exceeded 3 m. To eliminate inter‐observer bias, a single trained surveyor (SE) conducted all fish surveys using the standard UVC method (Jennings and Polunin 1995). At the start of each transect, he assessed the distance of visibility; then all observed individuals were identified to species level, counted, their lengths estimated, and assigned a perpendicular distance from the transect line. The UVC detailed methods are available in Tsemel et al. (2026).

### 2.3 Benthic and Environmental Measurements

Epibenthic cover was quantified using 25 × 25 cm photo‐quadrats taken every 2 m along the 25 m transect line. To reduce systematic bias and yield more accurate cover estimates, we fixed the camera to the quadrate, used a high‐resolution camera, and external flash lighting (Canon G1X with two external Ikelite DS51 flashes, at RAW capture mode).

All photos were uploaded to the ‘Coralnet’ website for point‐sampling analysis (Beijbom et al. 2015), and analyzed there. CoralNet suggested labels from our predefined taxonomic and functional label set for randomly allocated points within 16 blocks in each photo. All identifications were verified by human experts. Epibenthic coverage of the functional groups: coverage by sediment (soft substrate), bare rock, Algae, Porifera, Cnidaria, Bryozoa, and Ascidiacea was used in this work. Due to the rarity of Echinodermata sightings in our data (< 5), this group was excluded from analysis.

Habitat structural complexity was assessed at the beginning of the sampling campaign at all transect locations using a pressure sensor (Onset HOBO U20). We assessed the habitat's rugosity and profile curvature, following Lazarus and Belmaker's (2021) suggestions and their provided code. Curvature represents the average radius of the arcs at the local peaks and troughs of the profile. Rugosity was calculated as the ratio of chain‐to‐tape distance, dividing the true contour length of the benthic profile by its straight‐line horizontal displacement.

Surface‐water chlorophyll a was collected in triplicate 170 mL samples during each excursion and measured according to Mandalakis et al. (2017).

Temperature measurements at the maximum depth of each dive were obtained from the Dive computers (Shearwater Petrel2). CTD (conductivity, temperature, depth; SBE 19plusV2) profiles of turbidity (Wetlabs) and chlorophyll fluorescence (Turner Cyclops) were collected in spring 2023 to characterize the water‐column conditions associated with the visibility gradient observed along the coastline at the 25–30 m depth sites.

### 2.4 The Data Obtained

The epibenthic cover and structural rugosity (*N* = 377) analysis is resilient to sampling conditions due to external flash configurations, providing a comprehensive spatial matrix across all latitudes, depths, seasons, and years.

In contrast, the fish sampling procedure is heavily constrained by environmental limitations. Due to consistently poor visibility (< 3 m) at the southern sites, fewer data are available there (*N* = 317). Five fish transects had no fish observations; thus, the RDA analysis was based on *N* = 312. The 25–30 m depth strata comprise 133 transects, while the 10–45 m depth analysis of the Achziv and Sdot Yam sites comprises 271 transects. A detailed description of the full data used in our analyses is available in Table A1.

Habitat structural heterogeneity data were available for 90 of the transects and were used to assess their importance as a predictor. Since it did not significantly explain variance in habitat or fish (Tables A2, A3), we ran the RDA analyses on the complete datasets.

Because the sampling occurred during the transition seasons, when rapid seawater warming or cooling occurs, and there were weeks between visits to different sites, temporal temperature changes between sampling dates were larger than representative spatial differences (Ozer et al. 2022), and temperature could not be used as an explanatory variable. To validate that assumption, we tested our argument and found that the measured temperatures at the different sites and depths did not explain any of the variance in the epibenthic data (RDA Ordistep *p* > 0.05); therefore, we excluded temperature from further analyses.

### 2.5 Fish Abundance

To address potential bias in fish counts due to lower visibility at lower latitudes and depths, we took two approaches: the first was to fit detection functions with a correction for visibility distance (assessed before the census of each transect) for each species with > 60 observations; the second was to use standard abundances derived from observations within 3 m of the transect line (the minimum visibility distance at which census can take place) in a 6 m × 25 m belt‐transect.

To account for variation in visibility and detectability, we attempted to fit distance‐sampling detection functions (ds function of the ‘Distance’ library; Miller et al. 2019) using hazard‐rate and half‐normal distributions, with visibility as a covariate, for species with sufficient observations (> 60), a minimum according to (Buckland et al. 2015). Due to very few observations at the 0–1 m distances, we binned the data to 0–2 m, then every m with an 8 m truncation distance.

We used the Goodness‐of‐Fit (Chi‐squared) test to evaluate how well our best‐fitting chosen detection function fit the actual distribution of the observed distance data. The null hypothesis is that the model provides an acceptable fit (*p*‐value > 0.05). A second criterion was to ensure the curve matches the data by visually comparing the cumulative distribution function (CDF) results with the empirical CDF against a 45‐degree line. If any of these tests failed, that meant that the best‐fitting function failed to fit our data. The binned detection function results failed for all of the species (*p*‐value < 0.001, Table A2).

A second attempt to fit distance detection models based on diver‐reported distances, without binning, failed the Cramér‐von Mises (CvM) goodness‐of‐fit test for all but 3 species (*Chromis chromis, Siganus rivulatus*, and *Siganus luridus* ; CvM *p*‐value > 0.05), which failed at the fitted vs. empirical CDF visual comparison (Table A3, Figure A3).

Consequently, distance‐based density corrections were abandoned, and all primary analyses were conducted using standard abundances from the 6 m × 25 m belt‐transect.

### 2.6 Fish Biomass

Fish species were also assigned to trophic guilds based on diet information compiled from the literature and FishBase (Boettiger et al. 2012). Trophic categories included herbivores, macro‐invertivores, micro‐invertivores, planktivores, piscivores, and omnivores, based on the dataset used by Givan et al. (2017), which was expanded to include a distinction between micro‐ and macro‐invertivores, based on available information in FishBase (Froese and Pauly 1994).

Fish biomass was estimated from species‐specific individual fish lengths using the standard allometric length‐weight relationship, *W* = *a* * *L* ^b^. *L* is the estimated length of the fish assigned by the surveyor (in centimeters), *a* is the scaling factor (intercept), and b is the allometric growth parameter (slope). Both parameters are available from FishBase via the ‘rfishbase’ R package. (Froese and Pauly 1994; Boettiger et al. 2012).

We evaluated the distribution of the biomass data against Poisson and negative binomial distributions, which may be suitable for over‐dispersed ecological count data. The negative binomial distribution was selected for the final models as it best accommodated our data. This choice was used in a generalized linear model (manyglm function from the ‘mvabund’ package; Wang et al. 2012) to assess how environmental variables, season, and year affect fish abundance and biomass across latitudinal and depth scales. The sediment coverage has strong seasonality. For this analysis, we considered the extent of sediment coverage as an environmental explanatory variable, which reflects sedimentation. Separate models were fitted for the latitude‐only subset (*N* = 133) and the full depth‐inclusive dataset (*N* = 269).

### 2.7 Fish Diversity

Gamma fish diversity was quantified for each of the 25–30 m depth sites, using generalized Hill numbers proposed by Chao et al. (2019). Data rarefaction enables comparisons of fish communities across locations that were sampled with varying effort. It was carried out and plotted with bootstrapped confidence intervals as well as extrapolations using the ‘iNEXT’ function from the R package ‘iNEXT’ (Hsieh et al. 2016).

### 2.8 Data Processing

Data handling and processing were carried out using the following R packages: ‘readr’ (Wickham 2016; Roberts 2019; Wickham et al. 2019, 2023, 2024; Kassambara 2020, 2021; Müller 2020; Wickham et al. 2024), ‘plyr’ (Wickham 2011), ‘dplyr’ (Wickham et al. 2023), ‘reshape2’ (Wickham 2007), ‘tidyverse’ (Wickham et al. 2019), ‘stringr’ (Wickham 2019), and ‘here’ (Müller 2020). The figures were prepared in R with ‘ggplot2’ (Wickham 2016), ‘labdsv’ (Roberts 2019), and ‘ggpubr’ (Kassambara 2020).

When the fish census was canceled due to poor visibility (120 transects), we did not assess visibility distance. To prevent loss of the epibenthic transect data in these cases, we inserted a conservative dummy value of 2.5 m for visibility.

### 2.9 Statistical Analyses

Statistical analysis was carried out with ‘stats’ (R Core Team 2024) and ‘rstatix’ (Kassambara 2021). Pearson's correlation and the plotting of environmental variables were performed using the ‘corrplot’ function in the ‘Corrplot’ library (Murdoch and Chow 1996). Correlation values that were not significant (*p* < 0.05) are not displayed.

### 2.10 Multivariate Analysis

The habitat at the different sites was characterized by first applying a Hellinger transformation to the coverage data for the different benthic coverage types to reduce the influence of dominant coverage types, keep all data within one order of magnitude, and preserve differences between samples. Environmental parameters used to explain the RDA were z‐standardized, centered around a mean of 0, and scaled to have a standard deviation of 1 using ‘decostand’ and ‘apply’ functions. Prior to conducting the Redundancy Analysis (RDA), we evaluated potential multicollinearity among our environmental predictors (visibility, latitude, mean depth, rugosity, curvature, season, and sample year) by calculating Variance Inflation Factors (VIF). It was then modeled using Redundancy Analysis (RDA in ‘Vegan’ (Oksanen et al. 2022)) to identify the main drivers of community variance. We tested the significance of the explanatory variables in explaining the community variance using forward selection ANOVA, ‘ordistep’, and the significance of the RDA ordination axes was tested with permutation ANOVA tests ‘anova.cca’. Variance Inflation Factor (VIF) is the standard way to check multicollinearity among explanatory variables in RDA and was tested using ‘vif.cca’, all from the ‘vegan’ package in R (Oksanen et al. 2022).

This procedure was repeated when we investigated how much variance in log‐transformed fish abundance was explained by epibenthic coverage (RDA1), visibility, latitude, mean depth, rugosity, curvature, and heterogeneity indices. All predictors had VIF values below 3 (the Epibenthic RDA1 VIF was the highest at 2.9). After confirming that the standard conservative threshold of 5.0 was retained to ensure stable and interpretable canonical coefficients, we performed the RDA analysis and the following steps.

### 2.11 Artificial Intelligence Generated Content

In preparing this manuscript, we used large language models (ChatGPT‐4, Gemini, and Claude) to assist with drafting R code, analyzing the discussion, offering advice on rearranging the introduction and methods section, and suggesting a final paragraph. Grammarly was used for grammar and style corrections. All outputs were reviewed and verified by the authors, who are responsible for the content.

## 3 Results

### 3.1 Spatial Environmental Gradients

The southern sites, Ashdod and Ashkelon, were characterized by flocculated waters with low visibility (divers' report). Visibility was below 3 m in most Ashdod UVCs and during many surveys in the Spring season at Ashkelon. Turbidity was significantly higher at Ashkelon, the southernmost site, throughout the water column, and increased by approximately 200% from a 15 m depth to the seafloor. At Sharon, turbidity increased in the lower 3 m, and at Sdot Yam only in the deeper 2 m (Figure A1a).

Surface seawater Chlorophyll a concentration (normalized) decreased with increasing latitude (ANOVA, *F*_1,209_ = 7.33, *p* = 0.007). In Ashkelon, there was a decrease in the Chlorophyll a fluorescence from 15 m depth toward the seafloor (Figure A1b). The southern sites of Ashdod and Ashkelon were characterized by increased habitat curvature and rugosity (Figure A2).

Epibenthic cover varied strongly among sites, largely tracking the latitudinal gradient. At the northern sites (Achziv, Sdot Yam), algae dominated the substrate (72.7% and 71.3% mean cover, respectively), while at the southern sites (Ashdod, Ashkelon), bryozoans dominated instead (80.5% and 74.2%), and algae cover was low (8.8% and 5.8%). Sharon, the central site, was intermediate, with both algae (54.0%) and bryozoans (51.2%) prominent. Soft substrate covered a substantial share of the seafloor at every site (37%–49% on average) and was highest at the southern sites (Ashkelon, 49.0%; Ashdod, 43.0%). Ascidians, hydrozoans, sponges, hard corals, and serpulids each covered less than 15% on average at any site, although ascidians and hydrozoans were more prevalent at the southern than at the northern sites.

Seasonally, algae cover was significantly higher in spring than in fall (49.9% vs. 35.3% mean cover; paired Wilcoxon test across the nine site–depth combinations, *W* = 0.0, *p* = 0.004). Bryozoan and soft‐substrate cover showed the opposite pattern—higher in fall than spring—but this trend was not significant (*p* = 0.16 for both). Hydrozoan cover was also higher in spring at most sites, though the difference was not statistically significant (*p* = 0.098).

Pearson correlation analysis (Figure 2) reveals that depth negatively correlated with visibility (Pearson *R =* −0.27; Figure 2) and coverage by algae (*R* = −0.48; *p* < 0.05) and was positively correlated with all invertebrate groups: sponges, ascidians, bryozoa, hydrozoans, and hard corals (*R* = 0.67, 0.42, 0.38, 0.29, 0.24; *p* < 0.05 for all). Depth was positively correlated with sediment coverage (*R* = 0.38; *p* < 0.05).

**FIGURE 2:**
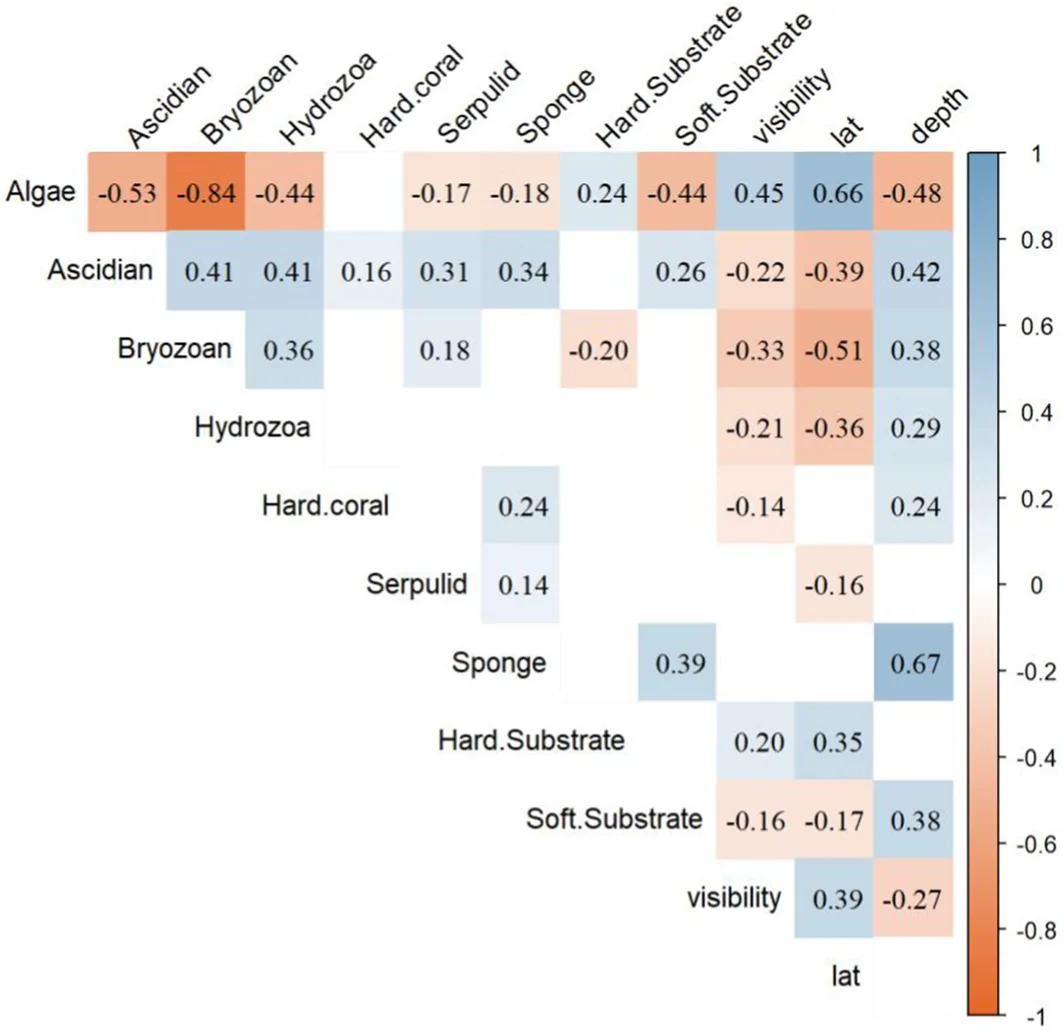
Pearson correlation matrix for epibenthic coverage groups and the environmental variables. Red represents a strong negative correlation, while blue represents a strong positive correlation. Values are presented for significant (*p* < 0.05) correlations only. Complete data set of 377 transects at all study sites and depths.

Algae coverage was negatively correlated with soft‐substrate coverage (*R* = −0.44; *p* < 0.05), whereas sponges and ascidians were positively correlated with sediment coverage (*R* = 0.39, 0.26). There was a negative correlation between algae and most other invertebrate groups, but the strongest was with bryozoan cover (*R* = −0.84; *p* < 0.05).

Higher latitudes were significantly positively correlated with greater visibility (*R* = 0.39, *p* < 0.05), increased algae (*R* = 0.66; *p* < 0.05), and bare rock coverage (*R* = 0.35; *p* < 0.05) but negatively correlated with less sediment cover (*R* = −0.21; *p* < 0.05). Invertebrates, however, were less likely to be found at higher latitudes (*R* = −0.51, −0.39, −0.36 for bryozoans, ascidians, and hydrozoans; *p* < 0.05 for all).

Across our study sites at different depths and latitudes, 55.5% of the epibenthic variance was explained by the RDA (Figure 3, Table 1). Latitude explained most of the epibenthic variance, followed by depth, season, distance of visibility, and year of survey (Table 1). There were no collinearity issues among these explanatory variables (all VIF values were below 2.2).

**FIGURE 3:**
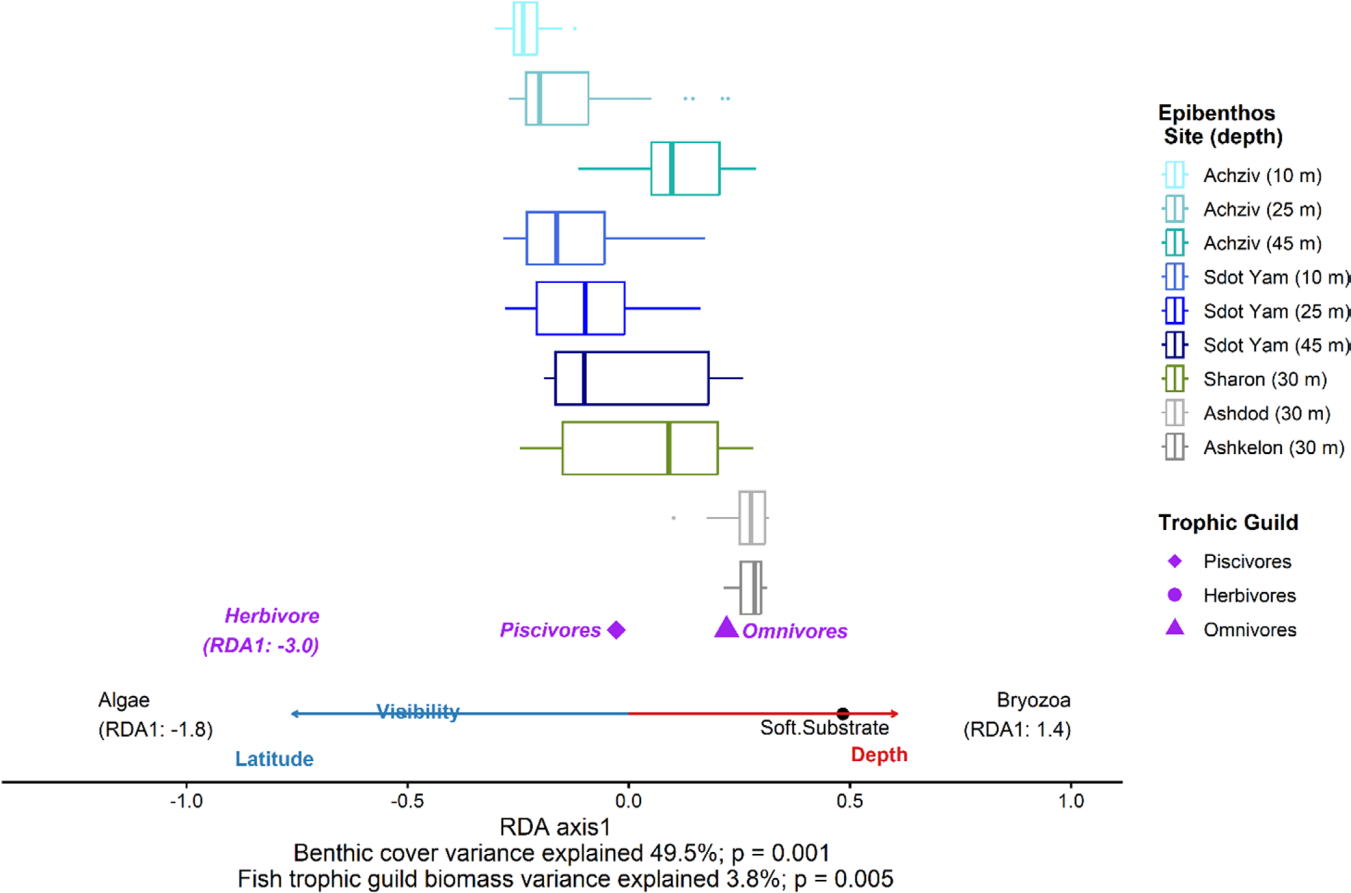
Redundancy analysis single‐axis (RDA1) plot of Hellinger‐transformed epibenthic grouped data (2017–2022), according to the sampling sites and depths (m), with standardized environmental parameters indicated by arrows. The RDA results are available in Table 1. The fish trophic guild biomass RDA results are overlaid in purple.

**TABLE 1:** Significant explanatory variables of the RDA Model of Hellinger‐transformed epibenthic grouped data (2017–2022) ~ depth + visibility + season + latitude + year, at all depths and sites.

RDA model	df	AIC	*F*	*p*
Visibility distance	1	−693	8.2	0.005
Year	5	−673	7.4	0.005
Season	1	−679	22.6	0.005
Depth	1	−614	95.9	0.005
Latitude	1	−585	133	0.005

Environmental conditions that correlated with latitude were the strongest predictors of benthic coverage types, causing a shift from algal to bryozoan dominance and increased sediment cover (Table 1, Figure 3), mirroring depth‐related changes within sites. Southern sites consistently exhibited more sedimentation and bryozoan coverage.

Differences in structural habitat complexity across depths and latitudes did not account for significant variation in epibenthic composition (Figure A2, Table A4).

### 3.2 The Fish Community

Visual surveys from 2017 to 2022 at the 25–30 m depth sites recorded 3859 fishes across 35 species and 20 families. The most abundant species were the schooling planktivore *Chromis chromis* (1384), the herbivore *Siganus rivulatus* (588), and the benthic micro‐invertivore *Coris julis* (405). Sdot Yam was the most abundant site (34.4 ± 32.3, mean ± SD) fish per transect, followed by Achziv (32.0 ± 32.0) and Ashdod (23.2 ± 22.0; *N* = 9), Sharon (17.8 ± 12.8), and Ashkelon (12.1 ± 22.0).

At the trophic guild level, the only true herbivores, the invasive rabbitfishes ( *Siganus rivulatus* and *S. luridus* ), were common at the northern sites, with 3.02 ± 7.06 individuals per 150 m^2^ at Achziv and 9.6 ± 24.61 per 150 m^2^ at Sdot Yam. At the central site of Sharon, only one observation of *S. rivulatus* had been recorded, and none had been observed at the southern sites. Herbivore and micro‐invertivore abundance were positively correlated with latitude (Negative binomial distribution, AIC = 418, coeff = 0.78, *p* = 0.02, and AIC = 450, coeff = 0.41, *p* = 0.01, respectively). The other trophic guilds did not show a latitudinal trend.

Due to low visibility in Ashkelon and Ashdod, fish surveys were often impossible, leading to highly uneven effort across excursions at the different sites. To overcome sampling effort bias and analyze the latitudinal aspect of the fish community (within 25–30 m depth strata), we used rarefaction, plotting the diversity vs. sampling effort (as depicted in Figure 4). Within the 25–30 m depth strata, at equal sampling effort (*N* = 9), Achziv and Sdot Yam exhibited the highest fish species richness, effective number of species, and number of dominant species, followed by Sharon and Ashkelon, with Ashdod exhibiting the lowest values. But due to the extremely low number of successful surveys, the gamma diversity at Ashdod cannot be interpreted differently from Ashkelon, the other southern site (Figure 4).

**FIGURE 4:**
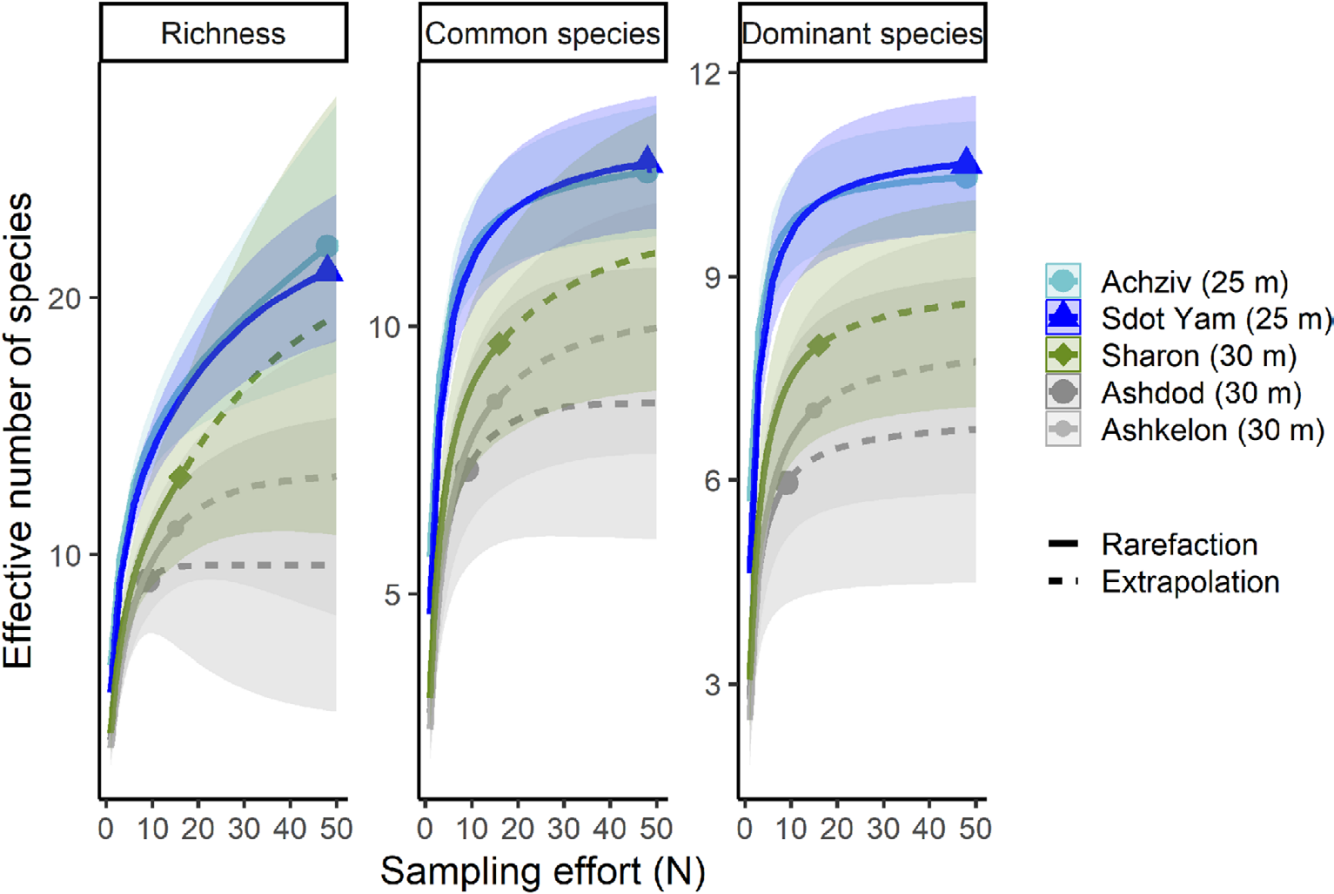
Rarefaction curves of measured and extrapolated fish gamma‐diversity at the 25–30 m depth sites. Left panel showing species richness, middle panel showing the effective number of common species (log of Shannon), and right panel showing the effective number of dominant species (inverse Simpson).

We found significantly higher species richness and the number of common species in the northern sites (Achziv + Sdot Yam) than in the southern sites (Sharon + Ashkelon; Wilcoxon paired test results with Bonferroni adjustment; Table A5).

Fish log‐transformed abundance RDA results (species_log ~ visibility + lat + mean_depth + Rugosity + Curvature + Epibenthos) on the 90‐transect dataset resulted in 23% of the variance explained, but the habitat heterogeneity variables were not significant predictors (Table A6). Exploring the complete‐case dataset of 312 transects' log of abundance resulted in 20% of the variance explained, with most of the variance explained by depth, followed by distance of visibility, latitude, and epibenthic coverage RDA1 (Table 2).

**TABLE 2:** Log‐transformed fish abundance RDA results (~ visibility + lat + depth_category + Epibenthos RDA1) 20% of the variance explained, Adjusted *R*‐square = 0.19.

	Df	Variance	*F*	Pr(>*F*)
Model	5	6.8	15.3	0.001***
Residual	305	27.0		
	Df	Variance	*F*	Pr(>*F*)
Visibility distance	1	1.1	12.1	0.001***
Latitude	1	0.6	6.3	0.001***
Depth category	2	4.6	26.0	0.001***
Epibenthic RDA1	1	0.5	5.9	0.001***
Residual	305	27.0		

*** *p* < 0.001.

### 3.3 Fish Biomass

Redundancy analysis of log‐transformed trophic guild biomass with visibility + lat + depth category + Epibenthos RDA as predictors results in 13% of the data variance explained, with latitude as the strongest predictor, followed closely by depth, the distance of visibility, and Epibenthic RDA1 (Table 3).

**TABLE 3:** Log‐transformed trophic group biomass RDA results (~ visibility + lat + depth category + Epibenthos RDA1). Adjusted *R*‐squared: 0.13.

	Df	Variance	*F*	Pr(>*F*)
Model	5	36.8	10.3	0.001***
Residual	300	214.5		
	Df	Variance	*F*	Pr(>*F*)
Distance of visibility	1	5.1	7.1	0.001***
Latitude	1	9.5	13.3	0.001***
Depth category	2	17.8	12.4	0.001***
Epibenthic RDA1	1	4.4	6.2	0.001***
Residual	300	214.5		

*** *p* < 0.001.

Linear regression results for the trophic group biomass vs. Epibenthic RDA1 resulted in very low (*R*‐squared 0.02–0.05), but significant decrease of Herbivore biomass with RDA1, and increase in piscivore and omnivore biomass with RDA1, as depicted in Figure 3.

GLM models show that the increase in herbivorous fish abundance with the Epibenthic RDA1 and with latitude was also significant for group biomass (Table 4, Models 1 and 5). Micro‐invertivore biomass was negatively associated with a shift away from an algal‐dominated epibenthos (RDA1; Model 3).

**TABLE 4:** Generalized linear model (based on negative binomial distribution) results for trophic group biomass (Herbivores, Micro‐invertivores).

Term	Model 1: Herbivore biomass ~ Latitude dataset	Model 2: Herbivore biomass ~ Depth dataset	Model 3: Micro‐invertivore biomass ~ Latitude dataset	Model 4: Micro‐invertivore biomass ~ Depth dataset
Intercept	−2.02	0.704	0.22	−0.078
visibility	0.237	0.155	0.131	0.231*
RDA1	−16.3**	−9.91**	−9.03**	−1.16
year2018	−0.716	0.113	1.37	0.636
year2019	0.88	0.242	2.12*	0.35
year2020	1.48	2.35**	1.51	1.28*
year2021	4.69*	2.14**	3.73**	1.84**
year2022	2.78	1.18	0.878	0.289
Season Spring	−2.04	NA	−1.90**	NA
N	133	269	133	269
Model test statistic	5.53	8.68	7.88	4.3
Residual deviance	69.2	213	115	213
AIC	503	1735	769	1413

Term	Model 5: Herbivore biomass ~ Latitude dataset	Model 6: Herbivore biomass ~ Depth dataset	Model 7: Micro‐invertivore biomass ~ Latitude dataset	Model 8: Micro‐invertivore biomass ~ Depth dataset
Intercept	3.34	3.59**	3.61	3.59**
latitude	1.62**	0.729*	0.245	−0.730**
depth	0.456	−1.75**	0.494	−0.296
Season Spring	−1.57	−0.661	−1.24**	−1.72**
N	133	269	133	269
Model test statistic	5.67	8.55	11.5	6.32
Residual deviance	70.6	214	117	214
AIC	505	1711	785	1399

*Note:* Predictors: horizontal distance of visibility, epibenthic community composition (RDA1), sample year, season, latitude (scaled), and depth (categoric). Significance codes: *p* < 0.001 ‘***’; *p* < 0.01 ‘**’; *p* < 0.05 ‘*’. Significant terms (*p* < 0.05) are highlighted in bold.

Similar to the trends observed in the latitudinal aspect, we observed decreasing herbivore biomass with increasing depth, and a shift away from an algal‐dominated epibenthos (RDA1) (Table 4, Models 6 and 2); Micro‐invertivore biomass declined significantly with RDA1 in the latitude data set and showed strong year‐by‐year and seasonal variability (Model 3). Invertivore biomass was positively associated with visibility in the depth data set (Model 4), while its relationship with the epibenthic RDA1 axis was non‐significant.

## 4 Discussion

The southeastern Mediterranean is influenced by the Nile Delta's recession, which releases nutrients, sediment, and possibly particulate matter carried by longshore currents (Figure 1 and Groom et al. 2005; D'Ortenzio and Ribera d'Alcalà 2009). The CTD profiles collected in 2023 align with long‐standing reports of flocculation and poor visibility at the southern sites, as well as with regional sediment size distributions (Almogi‐Labin et al. 2012). However, these profiles are inferential, since they are not co‐temporal with the 2017–2022 biological surveys. Our study suggests that community variation with distance from the Nile Delta and depth is consistent with light‐ and sediment‐driven shifts in the base of the benthic food web; co‐temporal optical and deposition measurements will be required to confirm causality.

The observed increase in surface Chlorophyll‐a concentrations at the southern sites (Figure 1) does not reflect benthic primary productivity, as algal coverage there is drastically reduced. The increased turbidity near the seabed (Figure A1a) appears to have a greater impact on this habitat. This snapshot data is supported by sediment size fractionation data along the Israeli coast (Almogi‐Labin et al. 2012), which is smallest at the southernmost Ashkelon (51% small‐sized particles, 5–30 μm), a size that, when mixed with larger particles, can be easily suspended in environments such as the one studied, which is exposed to wave action most of the year.

Epibenthic coverage is known to be significantly affected by environmental conditions, including sediment type and turbidity (Kennish 2001), as well as sediment load and light availability (Irving and Connell 2002; Hurlbut 1993). These factors are often interrelated (Maturo 1959; Irving and Connell 2002). These processes determine whether the base of the food web is dominated by invertebrates or algae (Irving and Connell 2002). While algal abundance decreases with sediment cover (Airoldi and Virgilio 1998; Irving and Connell 2002), bryozoans and other suspension‐feeding organisms proliferate in highly turbid, low‐light environments such as estuaries (Maturo 1959; Maughan 2001). Moreover, the coupled decline we observed across benthic primary producers, herbivorous fish, and micro‐invertivorous fish echoes their finding that biodiversity impacts intensify when multiple sediment‐impact pathways co‐occur, consistent with whole‐ecosystem disruption rather than a single‐taxon response (Magris and Ban 2019).

Shallow and deep habitats were unavailable at the southern sites; therefore, they were not sampled at Ashdod and Ashkelon, limiting direct tests of ‘mirroring’ at southern sites. Nevertheless, we cannot ignore the resemblance in community shifts across latitude and depth, in which we see the proliferation of bryozoans, ascidians, and hydrozoa at the expense of algae, not only between the south–north gradient and from shallow to deeper reefs but also at the central site (Sharon), which undergoes seasonal changes (Figure A4).

The shift from an epibenthic algae‐based food web to suspension‐feeding invertebrates at the upper‐mesophotic depths (25 to 30 m) was coupled with decreases in herbivorous and micro‐invertivorous fish abundance and increasing sediment coverage. This underscores a fundamental change in ecosystem functioning and is characteristic of the transition from shallow to mesophotic waters in our data, as well as in other studies of depth‐related processes (Kane and Tissot 2017; Moustaka et al. 2018; Cure et al. 2021). The reduced fish species richness and abundance, together with the absence of herbivores on the rock at the southern sites, are similar to those observed in lower mesophotic reefs compared to shallow reefs (Andradi‐Brown et al. 2016; Asher et al. 2017).

One effect of the scarcity of epibenthic algae cover in the southern sites is that the rabbitfishes (Siganidae), the dominant herbivores in our ecosystem, were missing altogether from our UVCs (Table A7). All other trophic groups were represented at all the southern sampling sites, albeit with a lower variety of representatives. Rabbitfish comprise the main diet of the most abundant grouper, the mottled grouper *Mycteroperca rubra* (Aronov and Goren 2008), which is less abundant in the southern range (Table A7), highlighting trophic cascades in these systems.

Demersal fishes have a pelagic larval phase lasting several weeks (Bradbury and Snelgrove 2001), during which dominant coastal currents can transport larvae across hundreds of kilometers (Manel et al. 2023), thereby theoretically homogenizing the regional species pool. Therefore, fish communities at this study's spatial scale (~160 km) are expected to be relatively homogeneous, given the high dispersal potential of most marine fish species. Yet despite the higher structural complexity of southern sites (Figures A2), known to positively influence fish richness and abundance (Letourneur 1996; García‐Charton and Pérez Ruzafa 1998; Harvey et al. 2013), and the higher abundance of fish eggs and larvae in the southern Levant Basin (Guerchon 2021), we still observe a significant decrease in fish abundance, richness, and diversity at the southern sites (Table A5). This decoupling between larval supply, habitat complexity, and fish abundance appears closely linked to strong environmental gradients in our data. Surface Chlorophyll‐a and sediment coverage decrease, while horizontal visibility, algal cover, and sediment grain size all increase northward, which may reflect the diminishing influence of the Nile Delta plume. These gradients likely shape post‐settlement conditions and habitat quality in ways that override the expected positive effects of structural complexity and larval availability at the southern sites.

These results directly contradict our initial hypothesis. We predicted that elevated surface productivity near the Nile Delta would produce an algae‐dominated epibenthic base and, consequently, higher fish abundance and diversity at the southern sites. Instead, we observed the opposite pattern at every level of the food web: reduced algal cover, fish species richness, and fish abundance, and the loss of the herbivore trophic level toward the Delta, while bryozoan‐dominated, sediment‐laden habitats prevailed. This reversal indicates that our original framing, in which nutrient enrichment was assumed to be the dominant process linking the Nile Delta plume to reef community structure, was too straightforward. Our results show that, within the depth range we surveyed, the light‐limiting and substrate‐altering effects of suspended sediment seem to override any stimulatory effect of the accompanying nutrients on the benthic primary producers that support herbivorous and micro‐invertivorous fishes. In other words, the Nile Delta plume seems to act here principally as an ocean‐darkening source rather than a productivity stimulator for the 25–30 m deep rocky reef community.

A previous study in this locality (Tsemel et al. 2026) shows that sites at northern depths of 25 m have the potential to provide a mesophotic refuge for shallower communities. The current results show that some of this resilience potential is compromised in the south due to the prevalence of unsuitable habitats there.

Ultimately, these findings reveal a concerning spatial constraint on suitable habitats within the Levant Basin, where environmental stressors mimic the limitations of the lower mesophotic zone at much shallower depths. This compression of the photic/mesophotic boundary at our southern sites reflects a pattern now documented globally: satellite‐derived estimates show that the photic zone has already darkened across roughly one‐fifth of the world's oceans over the past two decades, with coastal darkening attributed in part to rising sediment and nutrient loads (Davies and Smyth 2025). Our site‐level dataset, which pairs epibenthic, structural, and fish‐community data, offers a mechanistic, ground‐truthed complement to these remote‐sensing findings, showing concretely how such optical changes propagate into shifts in benthic community structure and trophic function.

The convergence of high turbidity, fine‐sediment deposition, and the resulting loss of algal‐driven productivity suggests that the southern Levantine coast may no longer function as a viable corridor for shallow‐water assemblages but rather as a restrictive filter. By decoupling structural complexity from biodiversity, these latitudinal and depth‐related gradients expose a critical vulnerability: the northern reefs' potential to serve as a climatic refuge is not a universal safeguard. Instead, as species are pushed from warming shallows into these functionally “squeezed” southern habitats, where the benthic food web is fundamentally altered, the ecosystem may reach a tipping point at which local extinctions outweigh colonization. This spatial shift in ecosystem functioning underscores that protecting Mediterranean biodiversity requires more than preserving physical structure; it requires maintaining the specific environmental windows that allow the development of the entire trophic hierarchy.

## 5 Conclusion

Our findings reveal a distinct coastal ecocline along the southeastern Mediterranean, where a transition from algae‐dominated to suspension‐feeder‐dominated epibenthos defines a major functional boundary. This benthic shift, associated with gradients in depth, latitude, sedimentation, and light attenuation, closely mirrors the transition observed in deeper mesophotic zones. The shared depth‐latitude gradient, acting in part through sedimentation and light attenuation, appears to be a major architect of fish community structure in this region, rather than structural complexity. This implies that the southern Levant may act not as a corridor but as a restrictive filter, trapping species between warming surface waters and a deep‐sea‐like habitat that lacks the conditions required to support higher trophic levels. These results align with recent evidence of global‐scale ocean darkening (Davies and Smyth 2025), suggesting that coastal systems worldwide facing increasing sediment loads and optical darkening may see similarly compressed, low‐productivity habitats emerge. Conserving marine biodiversity in such systems will require moving beyond the physical protection of reef structures to actively preserving the environmental conditions that sustain autotrophic food webs.

## Author Contributions

**Anat Tsemel:** conceptualization (lead), formal analysis (lead), visualization (lead), writing – original draft (lead), writing – review and editing (lead). **Hagai Nativ:** data curation (equal), methodology (equal). **Stephane Martinez:** data curation (equal), methodology (equal), writing – review and editing (supporting). **Shai Einbinder:** data curation (equal), funding acquisition (equal), investigation (equal), methodology (equal), resources (equal), validation (equal). **Ole Johannes Ringnander Sørensen:** writing – review and editing (supporting). **Yair Suari:** data curation (supporting), visualization (supporting), writing – review and editing (supporting). **Ido Izhaki:** formal analysis (supporting), supervision (lead), writing – review and editing (supporting). **Dan Tchernov:** conceptualization (lead), funding acquisition (equal), resources (equal), supervision (supporting).

## Funding

This work was supported by the Kadas Family Charitable Foundation and the Kahn Foundation.

## Ethics Statement

This work did not require any approvals, licenses, or permissions.

## Conflicts of Interest

The authors declare no conflicts of interest.

## Figures and Tables

**FIGURE A1:**
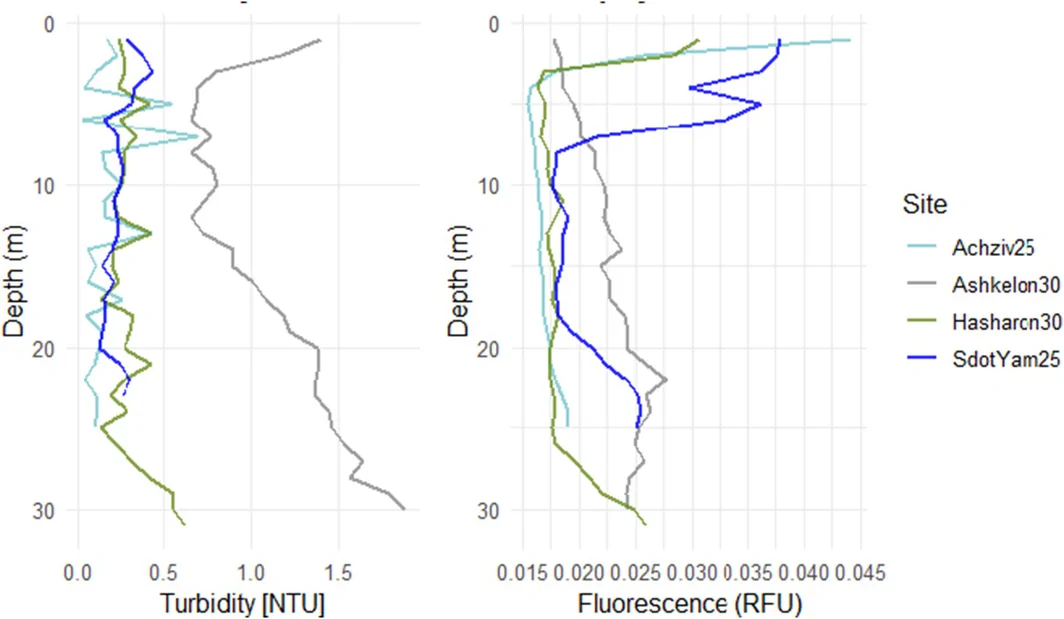
(a) Turbidity (b) fluorescence cast data as a proxy of Chl‐a at the sampling sites during the Spring of 2023.

**FIGURE A2:**
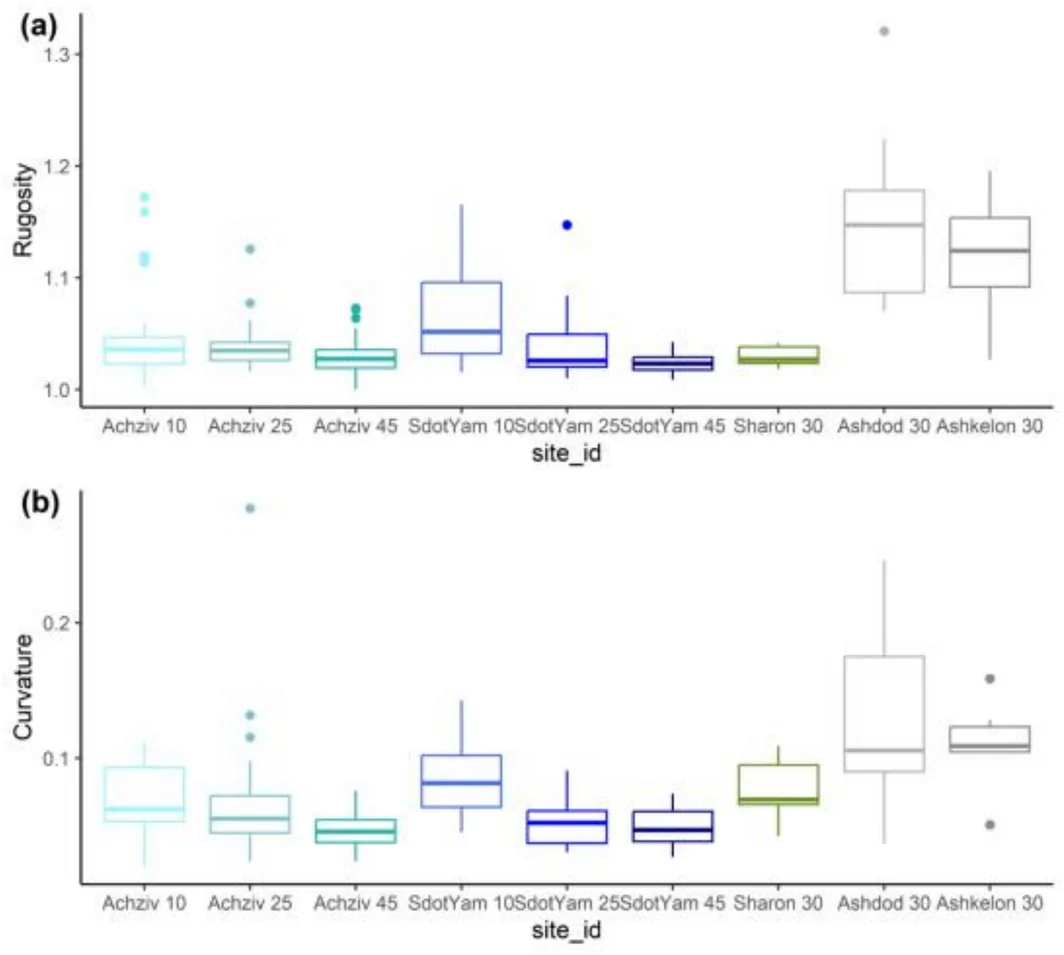
Habitat rugosity (a) and curvature (b) at the different study sites.

**FIGURE A3:**
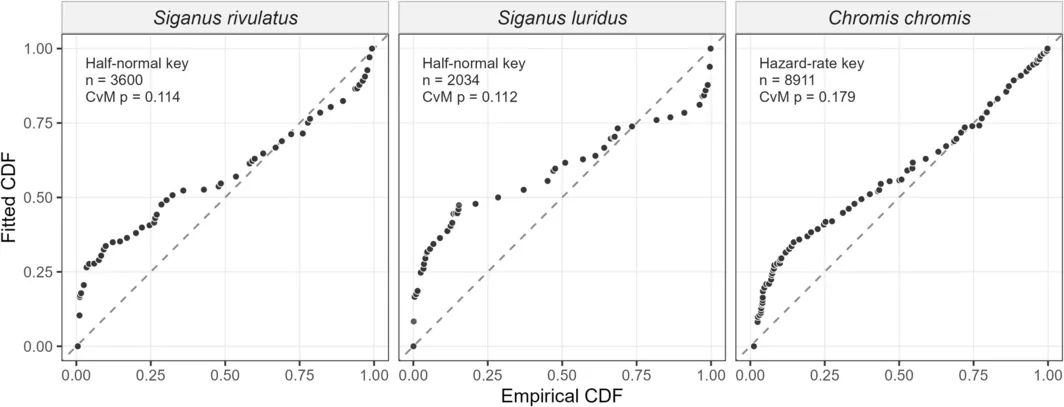
Cumulative distribution function fitted CDF results vs. the empirical CDF against a 45‐degree line. In a good fit, the points would fall on the 45‐degree diagonal line.

**FIGURE A4:**
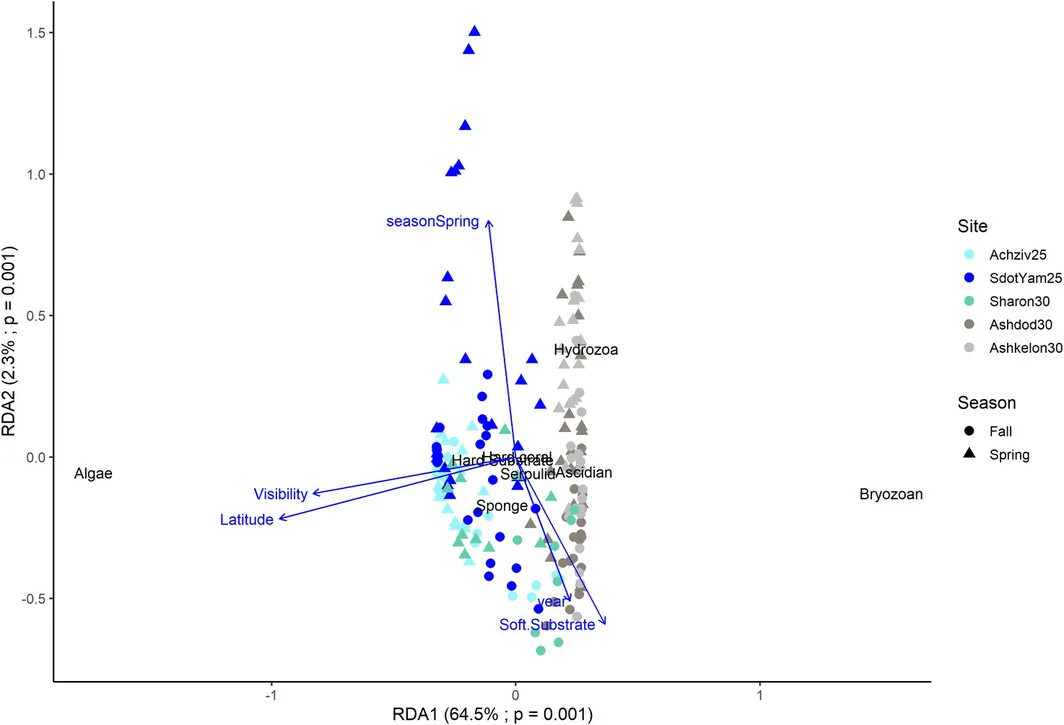
Redundancy analysis and ordination of epibenthic coverage at the 25–30 m depths across all sites, from 2017 to 2022, according to sampling sites and season, with environmental parameters as arrows.

## Data Availability

The data and codes are available in Zenodo: https://zenodo.org/records/21626001.

## Appendix A

### A.1 Sampling effort

**TABLE A1:** Data collected and analyzed in this study per year, season and site.

Site		Achziv			Sdot Yam			Sharon	Ashdod	Ashkelon	Data collected
Depth		10 m	25 m	45 m	10 m	25 m	45 m	30 m	30 m	30 m	
2017	Spring										Extracted chlorophyll
											Epibenthic point sampling
					3						Fish census
											Habitat heterogeneity
	Fall										Extracted chlorophyll
											Epibenthic point sampling
											Fish census
											Habitat heterogeneity
2018	Spring										Extracted chlorophyll
											Epibenthic point sampling
											Fish census
											Habitat heterogeneity
	Fall										Extracted chlorophyll
											Epibenthic point sampling
											Fish census
											Habitat heterogeneity
2019	Spring										Extracted chlorophyll
											Epibenthic point sampling
											Fish census
	Fall										Habitat heterogeneity
											Epibenthic point sampling
							2				Fish census
2020	Spring										Extracted chlorophyll
											Epibenthic point sampling
											Fish census
	Fall										Extracted chlorophyll
											Epibenthic point sampling
											Fish census
											Habitat heterogeneity
2021	Spring										Extracted chlorophyll
											Epibenthic point sampling
											Fish census
	Fall										Extracted chlorophyll
											Epibenthic point sampling
											Fish census
2022	Spring										Extracted chlorophyll
											Epibenthic point sampling
											Fish census
	Fall										Epibenthic point sampling
											Fish census
2023	Spring										CTD

*Note:* Habitat heterogeneity (grey), Fish census (orange), Epibenthic photogrammetry (pink), Extracted Chlorophyll (green), CTD, turbidity, and chlorophyll fluorescence (blue; at the bottom). The numbers in the table indicate fish sampling effort different than 4.

### Fish abundance

**TABLE A2:** Summary of the best‐fitting distance‐sampling detection functions and model parameters for fish species observed (*N* > 60) across depth and latitudinal gradients for binned distances: 0–2, 3, 4, 5, 6, 7, and 8.

Species	Best model	AIC	*N*.	Avg *p*	Avg *p* SE	Avg *p* CV	Chi‐squared *p‐*value
*Chromis chromis*	hr	31,148	8911	0.59	18.29	0.07	0.00E+00
*Coris julis*	hr	2748	943	0.41	2.87	0.07	0.00E+00
*Diplodus cervinus*	hn	381	98	0.58	14.65	0.69	0.00E+00
*Diplodus sargus*	hn	541	150	0.47	4.88	0.27	0.00E+00
*Diplodus vulgaris*	hn	171	52	0.38	10.00	0.56	1.13E‐05
*Epinephelus costae*	hn	1061	276	0.56	6.70	0.27	0.00E+00
*Epinephelus marginatus*	hn	1423	400	0.46	4.79	0.17	0.00E+00
*Mycteroperca rubra*	hn	3534	937	0.53	5.86	0.12	0.00E+00
*Parupeneus forsskali*	hn	2951	807	0.49	6.55	0.12	0.00E+00
*Pterois miles*	hn	710	433	0.16	331.05	0.87	0.00E+00
*Sardin sp*	hn	1331	531	0.26	68.39	0.24	0.00E+00
*Sargocentron rubrum*	hn	2671	792	0.41	58.82	0.55	0.00E+00
*Serranus cabrilla*	hn	559	225	0.26	3.27	0.11	1.73E‐04
*Siganus luridus*	hn	8168	2034	0.68	10.77	0.21	0.00E+00
*Siganus rivulatus*	hn	14,067	3600	0.61	14.99	0.12	0.00E+00
*Sparisoma cretense*	hr	842	265	0.50	6.53	0.18	0.00E+00
*Thalassoma pavo*	hr	5698	1985	0.44	4.90	0.08	0.00E+00
*Torquigener flavimaculosus*	hn	558	184	0.34	6.85	0.20	1.83E‐14

*Note:* Models were selected based on the lowest Akaike's Information Criterion (AIC). *N* indicates the number of independent observations used to fit each model. Average detection probability (Avg *p*), its standard error (Avg *p* SE), the coefficient of variation (Avg *p* CV), and the Cramér‐von Mises (CvM) goodness‐of‐fit test are presented to assess model parameter precision. In red Chi‐square *p*‐value ≤ 0.05.

Abbreviations: hr, hazard‐rate; hn, half‐normal.

**TABLE A3:** Summary of the best‐fitting distance‐sampling detection functions and model parameters for fish species observed across depth and latitudinal gradients for observer‐reported distances.

species	Best model	AIC	*N*	Avg *p*	Avg *p* SE	Avg *p* CV	CvM *p* value	Fitted cdf/Empirical cdf
*Chromis chromis*	hr	31,148	8911	0.59	18.29	0.07	1.79E‐01	Poor fit Figure A3
*Coris julis*	hr	2748	943	0.41	2.87	0.07	4.71E‐06	
*Diplodus cervinus*	hn	381	98	0.58	14.65	0.69	1.30E‐06	
*Diplodus sargus*	hn	541	150	0.47	4.88	0.27	4.84E‐07	
*Diplodus vulgaris*	hr	140	52	0.39	7.35	0.43	1.78E‐06	
*Epinephelus costae*	hn	1061	276	0.56	6.70	0.27	8.79E‐07	
*Epinephelus marginatus*	hn	1423	400	0.46	4.79	0.17	1.83E‐05	
*Mycteroperca rubra*	hn	3534	937	0.53	5.86	0.12	6.75E‐03	
*Parupeneus forsskali*	hn	2951	807	0.49	6.55	0.12	3.95E‐04	
*Sardin sp*	hn	1331	531	0.26	68.39	0.24	1.25E‐02	
*Sargocentron rubrum*	hn	2671	792	0.41	58.82	0.55	3.73E‐04	
*Serranus cabrilla*	hn	559	225	0.26	3.27	0.11	1.12E‐02	
*Siganus luridus*	hn	8168	2034	0.68	10.77	0.21	1.12E‐01	Poor fit Figure A3
*Siganus rivulatus*	hn	14,067	3600	0.61	14.99	0.12	1.14E‐01	Poor fit Figure A3
*Sparisoma cretense*	hr	842	265	0.50	6.53	0.18	2.41E‐06	
*Thalassoma pavo*	hr	5698	1985	0.44	4.90	0.08	5.43E‐03	
*Torquigener flavimaculosus*	hn	558	184	0.34	6.85	0.20	1.06E‐02	

*Note:* Models were selected based on the lowest Akaike's Information Criterion (AIC). *N* indicates the number of independent observations used to fit each model. Average detection probability (Avg *p*), its standard error (Avg *p* SE), the coefficient of variation (Avg *p* CV), and the Cramér‐von Mises (CvM) goodness‐of‐fit test are presented to assess model parameter precision. In green, *p*‐value > 0.05; in red, *p*‐value < 0.05. Lastly, the fitted cdf/empirical cdf (Figure A3) visual assessment is presented for the successful models marked green.

Abbreviations: hr, hazard‐rate; hn, half‐normal.

### A.2 Environmental variables

#### Habitat complexity

The differing curvature at North (Achziv 25 and Sdot Yam 25) to South (Sharon30 and ‘Ashkelon30’) at the sampling sites (Spearman *R* = −0.62, *p* < 0.05; Figure A2) did not significantly explain any of the epibenthic data variability, which was collected at the same time period (*n* = 102, 2017–2018). The RDA model used was epibenthic cover ~ visibility + latitude + soft substrate + rugosity + categorized depth. Ordistep() procedure found that the significant variables in the model are: latitude and depth category (Table A4).

**TABLE A4:** RDA Model for Hellinger transformed epibenthic cover ~ visibility + latitude + soft substrate + rugosity + categorized depth, the (Ordistep) resulting significant explanatory variables are presented.

RDA model	Df	AIC	*F*	*p*
Soft Substrate	1	−236.1	3.1	0.01
Depth	2	−202.4	22.5	0.005
Latitude	1	−195.5	51.9	0.005

**TABLE A5:** Wilcoxon paired test results with Bonferroni adjustment for fish species alpha diversity (Effective numbers *q* = 0, 1, and 2), and abundance at the different regions, North (Achziv + Sdot‐Yam) and South (Sharon + Ashdod +Ashkelon).

North vs. South: Effective diversity indices	*W* statistic	*P*	Adj. *p*
Richness	3135.5	< 0.001	**< 0.001**
Exponent of Shannon	2973.5	< 0.001	**< 0.001**
Inverse Simpson	2897.5	< 0.001	**< 0.001**
Abundance	2768.5	< 0.001	**< 0.001**

*Note:* Bold *p* values indicate significant differences in diversity between the Northern and Southern regions.

RDA model results for log‐transformed fish species abundance ~ visibility + lat + mean_depth + Rugosity + Curvature + Epibenthos explained 23% of the fish variance in 90 transects with complete data (Table A6).

**TABLE A6:** RDA model results for fish species log‐ transformed abundance ~ visibility + lat + mean_depth + Rugosity + Curvature + Epibenthos RDA1, data = env_scaled for abundance and trophic group biomass (g) vs. RDA1 of the environmental variables.

Term	Df	Variance	*F*	Pr(>*F*)
Distance of visibility	1	2.171	7.5916	0.001***
Latitude	1	0.723	2.5284	0.021*
Mean depth	1	2.7145	9.4921	0.001***
Rugosity	1	0.444	1.5525	0.147
Curvature	1	0.2281	0.7976	0.551
Epibenthic RDA1	1	0.8025	2.8064	0.004**
Residual	83	23.7356		

*Note:* ****p* < 0.001; ***p* < 0.01; **p* < 0.05.

**TABLE A7:** Fish species average biomass (± standard deviation) per 150 sqm at the rocky reef study sites during 2015–2022 according to trophic groups.

Diet	species	Achziv10	Achziv25	Achziv45	Ashdod30	Ashkelon30	SdotYam10	SdotYam25	SdotYam45	Sharon30
FC	*Argyrosomus regius*	—	—	325 ± 2255	—	55.6 ± 193	—	—	—	—
FC	*Epinephelus marginatus*	1023 ± 2079	683 ± 1546	2306 ± 4185	20.9 ± 46.1	78.8 ± 135	154 ± 272	399 ± 1308	97.8 ± 556	556 ± 1854
FC	*Muraena helena*	—	—	—	—	—	—	8.14 ± 36.5	10.5 ± 63.0	—
FC	*Mycteroperca rubra*	1123 ± 1953	1635 ± 3601	4424 ± 12,854	420 ± 755	76.2 ± 202	45.8 ± 123	1562 ± 5130	48.0 ± 165	1830 ± 5275
FC	*Pterois miles*	43.3 ± 191	68.1 ± 301	193 ± 643	—	4.03 ± 14.0	3.64 ± 19.4	42.0 ± 244	154 ± 578	13.3 ± 53.1
FC	*Sciaena umbra*	—	—	20,916 ± 52,908	—	—	—	—	—	—
FC	*Scomberomorus commerson*	—	—	—	—	—	—	50.0 ± 347	—	—
FC	*Seriola dumerili*	—	—	23.2 ± 161	—	—	—	155 ± 1071	—	—
FC	*Torpedo marmorata*	—	—	—	—	—	—	—	—	87.3 ± 349
H	*Siganus luridus*	288 ± 477	9.07 ± 34.3	—	—	—	51.1 ± 153	—	—	—
H	*Siganus rivulatus*	283 ± 595	13.6 ± 37.4	—	—	—	75.7 ± 167	17.3 ± 54.9	2.44 ± 6.59	0.040 ± 0.159
H	*Sparisoma cretense*	23.4 ± 130	1.81 ± 9.83	11.3 ± 68.7	—	—	—	2.49 ± 14.1	0.609 ± 1.57	—
Ma	*Balistes capriscus*	—	—	—	—	—	0.417 ± 2.89	—	—	—
Ma	*Coris julis*	8.40 ± 22.5	22.3 ± 30.6	8.74 ± 16.1	0.326 ± 0.978	—	0.583 ± 1.67	11.2 ± 15.8	8.97 ± 14.9	11.4 ± 13.1
Ma	*Dasyatis pastinaca*	—	377 ± 2612	—	—	—	—	—	—	—
Ma	*Diplodus cervinus*	—	—	8.10 ± 56.1	4.18 ± 8.73	81.3 ± 189	8.60 ± 28.1	—	—	—
Ma	*Diplodus sargus*	—	—	424 ± 2167	—	11.8 ± 40.9	15.0 ± 48.5	—	—	—
Ma	*Diplodus vulgaris*	—	—	22.7 ± 118	—	12.3 ± 25.9	0.555 ± 2.94	—	—	—
Ma	*Epinephelus costae*	414 ± 1040	186 ± 355	1756 ± 5233	260 ± 525	—	9.14 ± 46.9	517 ± 1649	2.77 ± 16.6	93.9 ± 376
Ma	*Pagrus auriga*	—	—	3.18 ± 22.0	—	—	—	—	—	—
Ma	*Pagrus caeruleostictus*	15.6 ± 107	—	0.256 ± 1.77	—	—	—	—	—	—
Ma	*Sargocentron rubrum*	17.6 ± 54.6	190 ± 195	—	12.8 ± 22.6	33.9 ± 92.0	5.48 ± 26.1	1.52 ± 9.21	—	21.7 ± 71.6
Ma	*Serranus cabrilla*	2.75 ± 11.0	6.22 ± 15.4	10.3 ± 21.5	1.49 ± 3.62	—	0.473 ± 2.29	5.70 ± 9.36	10.6 ± 12.3	4.94 ± 11.7
Ma	*Serranus scriba*	2.57 ± 6.57	0.929 ± 6.44	2.28 ± 15.8	—	—	0.393 ± 2.01	1.77 ± 5.69	—	—
Ma	*Taeniurops grabatus*	186 ± 1276	20.3 ± 140	659 ± 4567	—	—	—	—	—	—
Mi	*Acantholabrus palloni*	—	—	—	—	—	—	—	0.021 ± 0.128	—
Mi	*Parupeneus forsskali*	29.0 ± 80.2	8.21 ± 40.4	4.09 ± 15.4	—	0.192 ± 0.448	4.29 ± 11.3	11.9 ± 46.6	6.82 ± 26.2	12.9 ± 46.4
Mi	*Symphodus mediterraneus*	—	—	0.292 ± 2.02	—	—	—	0.436 ± 2.12	0.419 ± 2.06	—
Mi	*Symphodus tinca*	0.022 ± 0.151	—	—	—	—	0.537 ± 3.72	0.337 ± 2.18	0.189 ± 0.789	—
Mi	*Torquigener flavimaculosus*	2.74 ± 12.7	3.29 ± 10.1	0.901 ± 6.25	—	—	—	36.2 ± 65.5	8.00 ± 20.7	0.379 ± 1.51
O	*Scarus ghobban*	14.7 ± 83.7	25.4 ± 143	864 ± 5188	—	—	—	—	—	—
PK	*Chromis chromis*	94.8 ± 150	20.5 ± 43.7	35.5 ± 76.7	53.1 ± 57.4	11.4 ± 24.1	58.0 ± 67.7	28.8 ± 51.1	117 ± 140	23.8 ± 37.5

*Note:* Refer: Golani et al. 1983; Froese and Pauly 1994; Bariche 2006; Aronov and Goren 2008; Fanelli et al. 2015; Savva et al. 2020.
